# Quantitative Models of Phage-Antibiotic Combination Therapy

**DOI:** 10.1128/mSystems.00756-19

**Published:** 2020-02-04

**Authors:** Rogelio A. Rodriguez-Gonzalez, Chung Yin Leung, Benjamin K. Chan, Paul E. Turner, Joshua S. Weitz

**Affiliations:** a Interdisciplinary Graduate Program in Quantitative Biosciences, Georgia Institute of Technology, Atlanta, Georgia, USA; b School of Biological Sciences, Georgia Institute of Technology, Atlanta, Georgia, USA; c School of Physics, Georgia Institute of Technology, Atlanta, Georgia, USA; d Department of Ecology and Evolutionary Biology, Yale University, New Haven, Connecticut, USA; e Program in Microbiology, Yale School of Medicine, New Haven, Connecticut, USA; University of California, Irvine

**Keywords:** antimicrobial agents, bacteriophage therapy, bacteriophages, evolutionary biology, mathematical modeling, microbial ecology

## Abstract

The spread of multidrug-resistant (MDR) bacteria is a global public health crisis. Bacteriophage therapy (or “phage therapy”) constitutes a potential alternative approach to treat MDR infections. However, the effective use of phage therapy may be limited when phage-resistant bacterial mutants evolve and proliferate during treatment. Here, we develop a nonlinear population dynamics model of combination therapy that accounts for the system-level interactions between bacteria, phage, and antibiotics for *in vivo* application given an immune response against bacteria. We simulate the combination therapy model for two strains of Pseudomonas aeruginosa, one which is phage sensitive (and antibiotic resistant) and one which is antibiotic sensitive (and phage resistant). We find that combination therapy outperforms either phage or antibiotic alone and that therapeutic effectiveness is enhanced given interaction with innate immune responses. Notably, therapeutic success can be achieved even at subinhibitory concentrations of antibiotics, e.g., ciprofloxacin. These *in silico* findings provide further support to the nascent application of combination therapy to treat MDR bacterial infections, while highlighting the role of innate immunity in shaping therapeutic outcomes.

**IMPORTANCE** This work develops and analyzes a novel model of phage-antibiotic combination therapy, specifically adapted to an *in vivo* context. The objective is to explore the underlying basis for clinical application of combination therapy utilizing bacteriophage that target antibiotic efflux pumps in Pseudomonas aeruginosa. In doing so, the paper addresses three key questions. How robust is combination therapy to variation in the resistance profiles of pathogens? What is the role of immune responses in shaping therapeutic outcomes? What levels of phage and antibiotics are necessary for curative success? As we show, combination therapy outperforms either phage or antibiotic alone, and therapeutic effectiveness is enhanced given interaction with innate immune responses. Notably, therapeutic success can be achieved even at subinhibitory concentrations of antibiotic. These *in silico* findings provide further support to the nascent application of combination therapy to treat MDR bacterial infections, while highlighting the role of system-level feedbacks in shaping therapeutic outcomes.

## Abstract (precis)

This work develops and analyzes a novel model of phage-antibiotic combination therapy, specifically adapted to an *in vivo* context. The objective is to explore the underlying basis for clinical application of combination therapy utilizing bacteriophage that target antibiotic efflux pumps in Pseudomonas aeruginosa. In doing so, the paper addresses three key questions. How robust is combination therapy to variation in the resistance profiles of pathogens? What is the role of immune responses in shaping therapeutic outcomes? What levels of phage and antibiotics are necessary for curative success? As we show, combination therapy outperforms either phage or antibiotic alone, and therapeutic effectiveness is enhanced given interaction with innate immune responses. Notably, therapeutic success can be achieved even at subinhibitory concentrations of antibiotic. These *in silico* findings provide further support to the nascent application of combination therapy to treat MDR bacterial infections, while highlighting the role of system-level feedbacks in shaping therapeutic outcomes.

## INTRODUCTION

Multidrug-resistant (MDR) bacterial infections are a threat to global health. The World Health Organization (WHO) has reported that drug-resistant tuberculosis alone kills 250,000 people each year (1). Moreover, the United States Centers for Disease Control and Prevention (CDC) have reported 23,000 deaths each year attributed to drug-resistant pathogens, while their European counterparts have reported 25,000 deaths each year resulting from drug-resistant infections (2, 3). The WHO has identified and prioritized 12 MDR pathogens (1) in order to guide efforts toward the development of new antimicrobial treatments. The Gram-negative bacterium Pseudomonas aeruginosa has been identified as a critical priority by the WHO (1).

Bacterial viruses (i.e., bacteriophage or “phage”) represent an alternative approach to treat MDR bacterial infections. Phage lysis of bacterial cells can drastically change bacterial population densities. In doing so, phage exert a strong selection pressure on the bacterial population. As a result, phage-resistant mutants can appear and become dominant (4–6), whether via surface-based resistance (4, 7) or intracellular mechanisms (8). The possibility that phage therapy may select for phage-resistant bacterial mutants has increased interest in identifying strategies to combine phage with other therapeutics, e.g., antibiotics (4, 6, 7, 9–11). However, the realized outcomes of combination strategies are varied, ranging from successes *in vitro* (9) and *in vivo* (4, 11) to failure given *in vitro* settings (6).

In many cases, the mechanism(s) underlying potential phage-antibiotic interactions is unknown. There are exceptions; for example, Escherichia coli phage TLS and U136B infect the bacterium by attaching to the outer membrane protein TolC, which is part of the AcrAB-TolC efflux system (12, 13). It has been shown that phage TLS selects for *tolC* mutants that are hypersensitive to novobiocin (13). Moreover, TolC has been identified as a phage receptor in other Gram-negative pathogens (14, 15), giving further support to the combined use of phage and antibiotics. Similarly, the phage OMKO1 may be able to use multiple binding targets to infect P. aeruginosa, including the type IV pilus and the multidrug efflux pump MexAB/MexXY (7); both mechanisms can result in selection against drug resistance.

The ability of phage OMKO1 to select against drug resistance in P. aeruginosa suggests that a combination treatment of P. aeruginosa with phage OMKO1 and antibiotics can lead to an evolutionary tradeoff between phage and antibiotic resistance (7, 11). Phage-resistant mutants can show impairments of the multidrug efflux pump MexAB/MexXY (7), such as reduced functionality (or loss) of outer membrane porin M (OprM). This protein is part of the efflux pump complex and may act as a cell receptor of the phage OMKO1. Mutations in the gene encoding OprM can impair phage infection and restore the sensitivity to some classes of antibiotics, including ciprofloxacin (CP) (7). Such an evolutionary tradeoff may be leveraged clinically to limit the spread of resistance to phage and antibiotics. Therapeutic application of phage and antibiotics *in vivo* necessarily involves interactions with a new class of antimicrobial agents: effector cells within the immune system. Recent work has shown that phage and innate immune cells, specifically neutrophils, combine synergistically to clear otherwise fatal respiratory infections which neither phage nor the innate immune response could eliminate alone (5). This “immunophage synergy” is hypothesized to result from density-dependent feedback mechanisms (16). Phage lysis decreases bacterial densities such that the activated immune response can clear bacteria; without phage, the bacterial densities increase to sufficiently high levels that are outside the range of control by immune cells. However, the potential role of the innate immune response in the context of phage-antibiotic combination therapy remains largely unexplored.

Here, we develop and analyze a mathematical model of phage-antibiotic combination therapy that builds on the synergistic interactions between phage, antibiotic, and immune cells. In doing so, we extend a mathematical model of immunophage synergy (16) to take into account the pharmacodynamics and pharmacokinetics of an antibiotic, e.g., ciprofloxacin. At the core of the combination therapy model is its multiple-targeting approach: the phage target phage-sensitive (antibiotic-resistant) bacteria while the antibiotic targets phage-resistant (antibiotic-sensitive) mutants (7, 11). Critically, in this model we assume that immune effector cells can target both bacterial strains. As we show, combination therapy successfully clears infections insofar as immune responses are active. Our proof-of-principle systems-level model highlights the role of immune responses in developing and assessing the effectiveness of phage-based therapeutics for treatment of MDR pathogens, particularly MDR P. aeruginosa, which exhibit evolutionary tradeoffs.

## 

### Combination therapy model.

**FIG 1:**
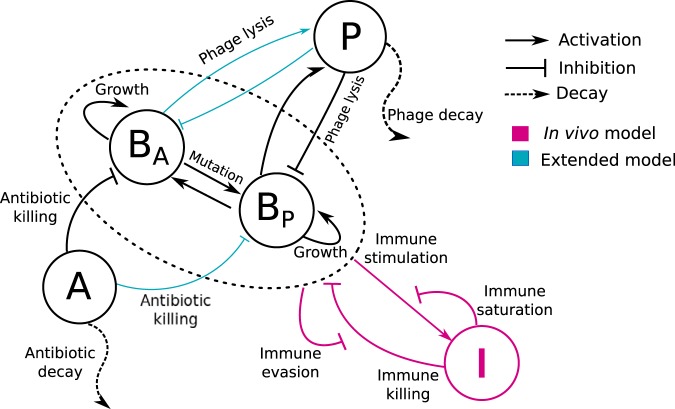
Schematic of the phage-antibiotic combination therapy model. Antibiotic-sensitive bacteria (*B_A_*) and phage-sensitive bacteria (*B_P_*) are targeted by antibiotic (A) and the phage (P), respectively. Host innate immune response interactions (pink arrows) are included in the *in vivo* model. Innate immunity (I) is activated by the presence of bacteria and attacks both bacterial strains. Furthermore, in model versions accounting for partial resistance (blue arrows), *B_A_* and *B_P_* are targeted by both antibiotic and phage but at quantitatively different levels.

We propose a combination therapy model consisting of a system of nonlinear, ordinary differential equations representing the interactions among bacteria, phage, antibiotics, and the innate immune system (see Fig. 1). Two strains of bacteria are included, one of which is phage sensitive (*B_P_*) and the other of which is antibiotic sensitive (*B_A_*). The strains *B_P_* and *B_A_* reproduce given limitation by a carrying capacity. *B_P_* is infected and lysed by phage (*P*) but resists the antibiotic, while the *B_A_* population is killed by the antibiotic but is resistant to phage (for an *in vitro* model of bacteriophage therapy with fully susceptible and resistant types, see reference 17). We do not consider double-resistant mutants in our model due to the evolutionary tradeoff between resistance against phage and antibiotics observed for P. aeruginosa (7). Phage replicate inside the host *B_P_* and decay in the environment. The antibiotic is administered at a constant concentration; then, it is metabolized and removed at a fixed rate. The population dynamics are governed by the following set of equations:

(1) $${\overset{˙}{B}}_{P}=\overset{B_{P}\;\text{growth}}{\overset{︷}{r_{P}B_{P}\left(1-\frac{B_{\text{tot}}}{K_{C}}\right)}}\;\overset{\text{Mutation}\;\text{to}\;B_{A}}{\overset{︷}{\left(1-\mu_{1}\right)}}+\overset{\text{Mutation}\;\text{from}\;B_{A}}{\overset{︷}{\mu_{2}r_{A}B_{A}\left(1-\frac{B_{\text{tot}}}{K_{C}}\right)}}\;-\;\overset{\text{Immune}\;\text{killing}}{\overset{︷}{\frac{\varepsilon IB_{P}}{1+\frac{B_{\text{tot}}}{K_{D}}}}}-\overset{\text{Lysis}}{\overset{︷}{B_{P}F(P)}}$$

(2) $${\overset{˙}{B}}_{A}=\overset{B_{A}\;\text{growth}}{\overset{︷}{r_{A}B_{A}\left(1-\frac{B_{\text{tot}}}{K_{C}}\right)}}\;\overset{\text{Mutation}\;\text{to}\;B_{P}}{\overset{︷}{\left(1-\mu_{2}\right)}}+\overset{\text{Mutation}\;\text{from}\;B_{P}}{\overset{︷}{\mu_{1}r_{P}B_{P}\left(1-\frac{B_{\text{tot}}}{K_{C}}\right)}}\;-\;\overset{\text{Immune}\;\text{killing}}{\overset{︷}{\frac{\varepsilon IB_{A}}{1+\frac{B_{\text{tot}}}{K_{D}}}}}-\overset{\text{Antibiotic}\;\text{killing}}{\overset{︷}{\kappa_{\text{kill}}\frac{A^{H}}{{\text{EC}}_{50}^{H}+A^{H}}B_{A},}}$$

(3) $$\overset{˙}{P}=\overset{\text{Viral release}}{\overset{︷}{\overset{˜}{\beta }B_{P}F(P)}}-\overset{\text{Decay}}{\overset{︷}{\omega P,}}$$

(4) $$\dot{I}=\overset{\text{Immune stimulation}}{\overset{︷}{\alpha I\left(1-\frac{I}{K_{I}}\right)\left(\frac{B_{\text{tot}}}{B_{\text{tot}}+K_{N}}\right)}},$$

(5) $$\dot{A}=\overset{\text{Antibiotic input}}{\overset{︷}{A_{I}}}-\overset{\text{Elimination}}{\overset{︷}{\theta A}}$$

In this model, phage-sensitive bacteria grow at a maximum rate *r_P_*, while antibiotic-sensitive bacteria (*B_A_*) grow at a maximum rate *r_A_*. The total bacterial density, *B*_tot_ = *B_P_* + *B_A_*, is limited by the carrying capacity *K_C_*. Phage infect and lyse *B_P_* bacteria at a rate *F*(*P*). Antibiotic killing is approximated by a Hill function with the nonlinear coefficient (*H*) (18–21). The maximum antibiotic killing rate is κ_kill_, while EC_50_ is the concentration of the antibiotic, here considered ciprofloxacin, at which the antibiotic effect is half the maximum. Phage *P* replicate with a burst size $\text{β}=\tilde{\beta }+1$ and decay at a rate ω. We assume that antibiotic dynamics are relatively fast and use a quasi-steady-state approximation of A* = *A_I_*/0.

When simulating an *in vivo* scenario, the host innate immune response, *I*, is activated by the presence of bacteria and increases with a maximum rate α. *K_N_* is a half-saturation constant, i.e., the bacterial density at which the growth rate of the immune response is half its maximum. Bacteria grow and are killed by the innate immunity with a maximum killing rate ε. However, at high bacterial concentration bacteria can evade the immune response and reduce the immune killing efficiency (5, 16).

Our model uses an implicit representation of spatial dynamics through different functional forms of phage-bacterium interactions [*F*(*P*)]. As such, we do not explicitly model the spatial dynamics of individual components. The model considers three modalities of phage infection, *F*(*P*): linear, heterogeneous mixing (5, 22), and phage saturation (5). The linear phage infection modality assumes a well-mixed environment, where phage easily encounter and infect bacteria, so the infection rate *F*(*P*) = ϕ*P* is proportional to the phage density, where ϕ is the linear adsorption rate. The heterogeneous mixing model accounts for spatial heterogeneity, $F(P)=\tilde{\phi }P^{\gamma }$, where $\tilde{\phi }$ is the nonlinear adsorption rate and *γ* < 1 is the power-law exponent. The third modality assumes that at high phage density multiple phage particles adsorb to a single bacterium so that phage infection follows a saturating Hill function,

$$F(P)=\frac{\phi P}{1+\frac{P}{P_{C}}}$$

Here, *ϕ* is the adsorption rate and *P_C_* is the phage density at which the infection rate is half saturated.

Note that in later stages, we consider an “extended” combination therapy model (Fig. 1 [blue arrows]) in which bacterial strains are sensitive to both phage and antibiotic in quantitatively distinct levels. The full set of equations for this extension is found in the supplemental material. In addition, a full description of parameter choices is given in Materials and Methods.

## RESULTS

### Differential outcomes of single-phage therapy.

**FIG 2:**
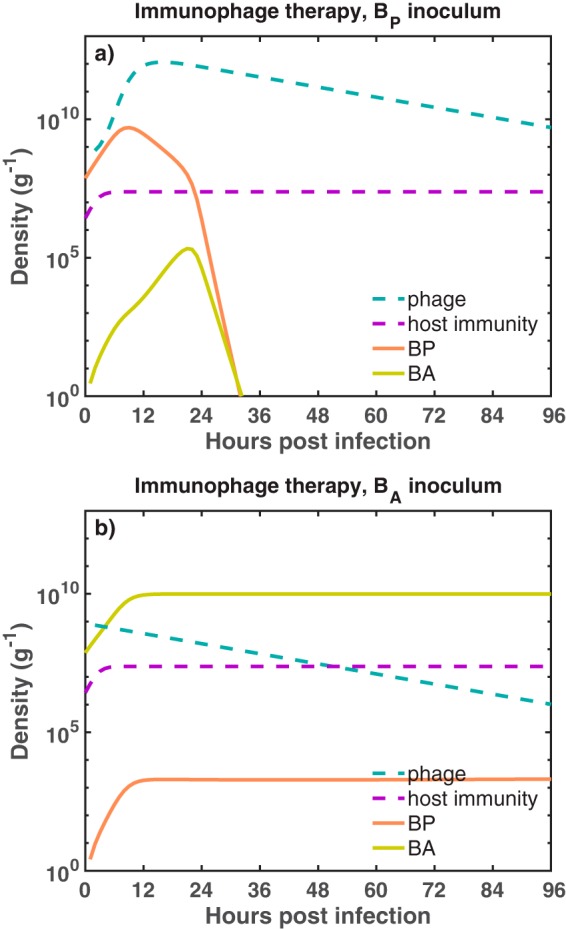
Dynamics of the immunophage therapy model against two different bacterial inocula. We simulate the phage therapy model developed in reference 16 against two infection settings. In the first infection setting (a), a phage-sensitive bacterial inoculum, *B_P_* (orange solid line), is challenged with phage (blue dashed line) inside an immunocompetent host. In the second scenario (b), antibiotic-sensitive bacteria, *B_A_* (green solid line), are challenged with phage in the presence of an active immune response (purple dashed line). The initial bacterial density and the initial phage density are *B*_0_ = 7.4 × 10^7^ CFU/g and *P*_0_ = 7.4 × 10^8^ PFU/g, respectively. For the simulation, we use a heterogeneous mixing model as a functional form of phage infection. The growth rates of *B_P_* and *B_A_* are *r_P_* = 0.75 h^−1^ and *r_A_* = 0.67 h^−1^, respectively. Simulation run is 96 h with phage being administered 2 h after the infection. The bacterial carrying capacity is *K_C_* = 10^10^ CFU/g.

We begin by exploring the dynamics arising from adding a single phage type at a density of 7.4 × 10^8^ PFU/g 2 h after infections caused by either phage-sensitive or phage-resistant bacteria (Fig. 2). When the infection is caused by a phage-sensitive bacterium (*B_P_* = 7.4 × 10^7^ CFU/g), phage lysis reduced *B_P_* density to the point where the immune response alone could control this bacterial population. Despite the emergence of phage-resistant mutants (*B_A_*), total bacterial population remained low and the innate immunity effectively controlled the infection. On the other hand, when the infection was caused by phage-resistant mutants (*B_A_* = 7.4 × 10^7^ CFU/g), the phage could not target *B_A_*, so the bacterial population grew unimpeded. The immune response was overwhelmed by the rapid growth of *B_A_*, which then reached a density of ∼10^10^ CFU/g after 12 h (Fig. 2b), leading to a persistent infection despite an activated immune response (similar to the outcomes described in reference 16).

This initial analysis illustrates how therapeutic outcomes given application of a single phage type may be strongly dependent on the initial bacterial inoculum. As expected, single-phage therapy fails to clear the infection when the bacterial inoculum is mistargeted (Fig. 2b). In the next section, we evaluate infection dynamics in response to the combined application of phage and antibiotics—similar to that in multiple *in vitro* and *in vivo* studies of phage-antibiotic treatment of MDR P. aeruginosa (7, 11).

### Phage-antibiotic therapy treatment dynamics in immunocompetent hosts.

**FIG 3:**
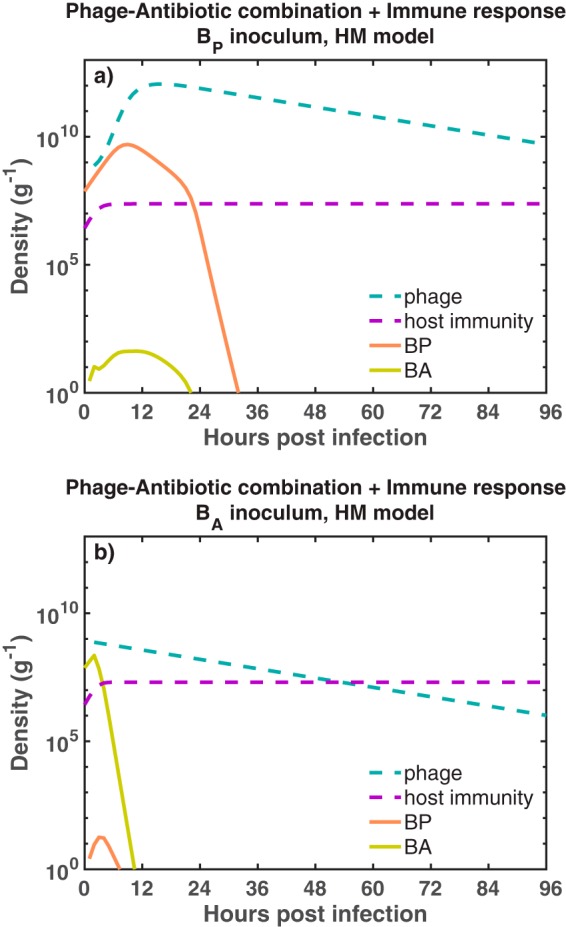
Outcomes of the phage-antibiotic combination therapy model for two different infection settings. We simulate the combined effects of phage and antibiotics in an immunocompetent host infected with phage-sensitive bacteria (a), *B_P_* (orange solid line). In panel b, the host is infected with antibiotic-sensitive bacteria, *B_A_* (green solid line). The dynamics of the phage (blue dashed line) and innate immunity (purple dashed line) are shown for each infection setting. Initial bacterial density and phage density are *B*_0_ = 7.4 × 10^7^ CFU/g and *P*_0_ = 7.4 × 10^8^ PFU/g, respectively. For the simulation, we use a heterogeneous mixing model as a functional form of phage infection. The simulation run is 96 h (4 days). Antibiotic and phage are administered 2 h after the beginning infection. Ciprofloxacin is maintained at a constant concentration of 0.0350* *μg/ml during the simulation. The carrying capacity of the bacteria is *K_C_* = 10^10^ CFU/g.

We simulated the combined effects of phage (7.4 × 10^8^ PFU/g) and antibiotics (assuming 2.5× MIC of ciprofloxacin for the *B_A_* strain) in two different infection settings. First, when an immunocompetent host was infected with phage-sensitive bacteria, the infection was cleared before ∼36 h due to the combined killing effect of phage, antibiotic, and innate immunity. The dominant bacterial population, *B_P_*, was targeted by the phage while the antibiotic targeted *B_A_*. The combined effects of phage and antibiotic reduced total bacterial density to the point where innate immunity eliminated the bacterial infection. Second, when the host was infected with antibiotic-sensitive bacteria, the pathogen was cleared (before ∼12 h) due to the combined effect of phage, antibiotic, and innate immunity. The antibiotic facilitated the decrease of *B_A_* while phage kept the *B_P_* concentration low, easing the innate immunity control over the infection. The resulting infection clearance in the phage-resistant case (Fig. 3b) stands in stark contrast to the previous outcome of the single-phage therapy model (Fig. 2b). Overall, the results suggest that a curative outcome is possible when phage are combined with antibiotics in an immunocompetent host—even when the phage is initially mistargeted to the dominant bacterial strain. The results hold for different functional forms of phage-bacterium interactions *F*(*P*) (see Fig. S1 in the supplemental material). However, what remains unclear is the extent to which successful treatment is driven by phage and antibiotics alone or, in part, because of the synergistic interactions with the innate immune response.

10.1128/mSystems.00756-19.3 FIG S1 Bacterial dynamics given different functional forms of phage infection and the presence of the host immune response. We simulate bacterial growth for 96 h in exposure to phage (blue dashed line), antibiotics (at a fixed level, not displayed), and the host immune response (purple dashed line). The combined therapy supplemented with the host immune response is tested against two different bacterial inocula. The first inoculum consisted of exclusively phage-sensitive bacteria (a to c), *B_P_* (orange solid line). The second inoculum consisted of antibiotic-sensitive bacteria (d to f), *B_A_* (green solid line). Additionally, we test three different models of phage infection, heterogeneous mixing (a and d), phage saturation (b and e), and linear infection (c and f). The initial bacterial density and phage density are *B*_0_ = 7.4 × 10^7^ CFU/g and *P*_0_ = 7.4 × 10^8^ PFU/g, respectively. Ciprofloxacin is maintained at a constant concentration of 2.5× MIC (i.e., 0.0350 μg/ml) during the simulations. Download FIG S1, PDF file, 0.1 MB. Copyright © 2020 Rodriguez-Gonzalez et al. 2020 Rodriguez-Gonzalez et al. This content is distributed under the terms of the Creative Commons Attribution 4.0 International license.

### Phage-antibiotic combination therapy requires innate immunity to robustly clear the pathogen.

In this section, we assess the dependency of combination therapy on the immune response. To do so, we evaluate the combination therapy while setting *I *= 0. This is meant to mimic conditions of severe immunodeficiency. In order to further assess outcomes, we also consider multiple functional forms for phage-bacterium interactions—including the phage-saturation, heterogeneous mixing, and linear infection models (see Materials and Methods for more details).

**FIG 4:**
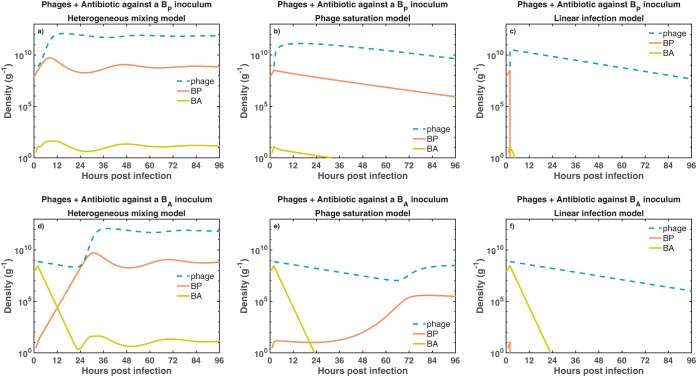
Bacterial dynamics given joint exposure to phage and antibiotic. We simulate bacterial growth for 96 h in exposure to phage (blue dashed line) and antibiotic (data not shown) added 2 h after the beginning of the inoculation. The combination of phage and antibiotic is tested against two different bacterial inocula. The first inoculum consisted of exclusively phage-sensitive bacteria (a to c), *B_P_* (orange solid line). The second inoculum consisted of antibiotic-sensitive bacteria (d to f), *B_A_* (green solid line). Additionally, we test three different models of phage infection, heterogeneous mixing (a and d), phage saturation (b and e), and linear infection (c and f). The initial bacterial density and phage density are *B*_0_ = 7.4 × 10^7^ CFU/g and *P*_0_ = 7.4 × 10^8^ PFU/g, respectively. Ciprofloxacin is maintained at a constant concentration of 2.5× MIC (i.e., 0.0350 μg/ml) during the simulations.

First, when a phage-sensitive bacterial inoculum was challenged with the combination therapy, the pathogen persisted in two of three infection models. Bacteria persist in the heterogeneous mixing (HM) (Fig. 4a) and phage saturation (PS) (Fig. 4b) models, while the combination of phage and antibiotic successfully eliminates the bacterial population in the linear infection (LI) model (Fig. 4c). Although the combination of phage and antibiotic did not eliminate the bacterial population in the HM and PS models, the combination strategy still reduced the bacterial concentration relative to the carrying capacity (*K_C_* = 10^10^ CFU/g).

Second, when an antibiotic-sensitive bacterial inoculum was challenged with phage and antibiotic, bacteria persisted in two of three infection models, similarly to the previous phage-sensitive case. Bacteria persist in the HM (Fig. 4d) and PS (Fig. 4e) models, while bacterial population is eliminated in the LI model (Fig. 4f). Inclusion of antibiotics facilitated a decrease in *B_A_* and the spread of *B_P_*, leading to coexistence between bacteria and phage. Furthermore, the elimination of bacteria in the LI model took longer (∼24 h) than in the previous phage-sensitive case.

The outcomes of the combination therapy model suggest that, in the absence of innate immunity, infection clearance is not achieved in two of three phage infection models. Pathogen clearance is achieved in only the linear infection case, that is, when we assume a well-mixed environment. On the other hand, when we assume spatial heterogeneity or phage saturation, a coexistence state between phage and bacteria arises from the tripartite dynamics between phage, bacteria, and antibiotic. Such a coexistence state is inconsistent with the expected antimicrobial effect of the combination therapy (7) and points to a potentially unrealized role of the immune response in the effectiveness of phage-antibiotic combination therapy.

### Outcomes of the combination therapy model are robust to the bacterial composition of the inoculum and the concentration of antibiotic.

Thus far, we have simulated two extreme infection inoculum scenarios involving exclusively phage-sensitive bacteria or exclusively antibiotic-sensitive bacteria. Next, we consider the effects of combination therapy on mixed bacterial inoculum containing both *B_P_* and *B_A_*. To do so, we performed a robustness analysis of four (*in silico*) therapy models, i.e., antibiotic-only, antibiotic-innate immunity, phage-antibiotic, and phage-antibiotic combination in the presence of innate immunity. For each model, we varied the concentration of the antibiotic and the bacterial composition of the inoculum. Outcomes from the different therapeutics are consistent with previous results obtained using a fixed set of initial conditions (Table 1). We find that model outcomes are robust to variations in the initial conditions (i.e., inoculum composition and concentration of ciprofloxacin).

**TABLE 1:** Summary of therapeutic outcomes given a combination of antibiotics (*A*), phage (*P*), and immunity (*I*)^*a*^

Treatment			Outcome
A	P	I	
1	0	0	Infection via *B_P_* proliferation
1	0	1	Infection via *B_P_* proliferation
1	1	0	Infection via *B_P_* coexistence with phage
1	1	1	Curative

a The presence or absence of different antimicrobial agents is represented with 1 or 0, respectively.

**FIG 5:**
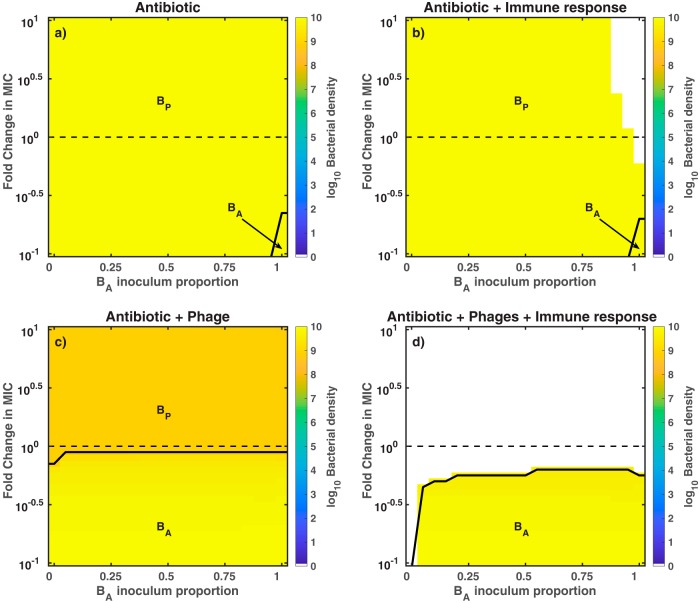
Outcomes of the robustness analysis for different antimicrobial strategies. We simulate the exposure of bacteria to different antimicrobial strategies, such as antibiotic-only (a), antibiotic plus innate immunity (b), phage plus antibiotic (c), and phage-antibiotic combination in the presence of innate immunity (d). The heatmaps show the bacterial density at 96 h postinfection. Colored regions represent bacterial persistence (e.g., orange areas for ∼10^9^ CFU/g and bright yellow areas for ∼10^10^ CFU/g), while the white regions represent pathogen clearance. We vary the concentration of ciprofloxacin (MIC = 0.014* *μg/ml), ranging from 0.1× MIC (0.0014* μ*g/ml) to 10× MIC (0.14* μ*g/ml), and the bacterial composition of the inoculum, ranging from 100% phage-sensitive bacteria (0% *B_A_*) to 100% antibiotic-sensitive bacteria (100% *B_A_*). Initial bacterial density and phage density (c and d) are *B*_0_ = 7.4 × 10^7^ CFU/g and *P*_0_ = 7.4 × 10^8^ PFU/g, respectively. Phage and antibiotic are administered 2 h after the beginning of the infection.

First, we evaluated the killing effect of the antibiotic against mixed bacterial inoculum. We find that the pathogen persisted (∼10^10^ CFU/g) for all different inoculum and concentrations of antibiotic. The antibiotic targeted *B_A_* while *B_P_* grew unimpeded in the absence of phage, such that *B_P_* predominated after 96 h. In contrast, antibiotics and innate immunity (Fig. 5b) could eliminate bacterial inoculum with high percentages of antibiotic-sensitive bacteria (>90% of *B_A_*). During this scenario, the low percentages of *B_P_* coupled with the antibiotic killing of *B_A_* facilitated the immune clearance of the infection. Furthermore, pathogen clearance was observed even for subinhibitory concentrations of ciprofloxacin. As is apparent, the antibiotic on its own cannot clear the infection, and therapeutic outcomes are only modestly improved in a narrow region of inoculum space.

Second, we assessed the effects of combining antibiotics with phage against mixed bacterial inoculum. The phage-antibiotic combination strategy failed to clear the infection for all combinations of initial conditions, consistent with the infection scenarios of the above section. Nonetheless, bacterial concentration was ∼10 times smaller due to phage killing (orange area in Fig. 5c) than the bacterial concentration from the antibiotic-only therapy (bright yellow area of Fig. 5a). After 96 h of combined treatment, the phage-sensitive population was predominant at above MIC levels while antibiotic-sensitive bacteria populated the sub-MIC levels (Fig. S2 and S3 show the effects of different antibiotic levels on the bacterial dynamics). In contrast, a robust pathogen clearance was achieved when the phage-antibiotic combination strategy was supplemented with active innate immunity (Fig. 5d). Note that even partially effective immune responses can still be sufficient to achieve infection clearance (Fig. S4). Overall, the synergistic interactions between phage, antibiotic, and innate immunity led to clearance of the infection for the majority of initial conditions. The clearance region even spanned subinhibitory concentrations of ciprofloxacin.

10.1128/mSystems.00756-19.4 FIG S2 Bacterial dynamics given exposure to low levels of antibiotics. We simulate the effects of combination therapy plus innate immunity on inocula with nontrivial levels of *B_A_*. First, an inoculum composed of 95% *B_P_* and 5% *B_A_* is treated with phage and different levels of antibiotic, 0, 0.001×, and 0.01× MIC (a, b, and c, respectively). The same treatment is applied for an inoculum composed of 80% *B_P_* and 20% *B_A_* (d, e, and f, respectively). Initial bacterial and phage density are *B*_0_ = 7.4 × 10^7^ CFU/g and *P*_0_ = 7.4 × 10^8^ PFU/g. Phage and antibiotic are administered 2 h after infection. The simulation run was 96 h (4 days). Download FIG S2, PDF file, 0.1 MB. Copyright © 2020 Rodriguez-Gonzalez et al. 2020 Rodriguez-Gonzalez et al. This content is distributed under the terms of the Creative Commons Attribution 4.0 International license.

10.1128/mSystems.00756-19.5 FIG S3 Accounting for variations in the concentration of antibiotic, from sub-MIC to MIC levels. We choose a particular inoculum composition (red boxes, 50% *B_P_* and 50% *B_A_*) from our heatmap and zoom in at the dynamics level. Bacterial dynamics correspond to different antibiotic levels: 1×, 0.5×, and 0.1× MIC levels (a, b, and c, respectively). The colored areas on the heatmap indicate bacterial presence while the white areas indicate infection clearance after 96 h of treatment. Phage and antibiotic are administered 2 h after infection. Initial bacterial and phage density, *B*_0_ = 7.4 × 10^7^ CFU/g and *P*_0_ = 7.4 × 10^8^ PFU/g, respectively. Download FIG S3, PDF file, 0.03 MB. Copyright © 2020 Rodriguez-Gonzalez et al. 2020 Rodriguez-Gonzalez et al. This content is distributed under the terms of the Creative Commons Attribution 4.0 International license.

10.1128/mSystems.00756-19.6 FIG S4 Bacterial density after 96 h of combined treatment with intermediate immune response levels. We extend our robustness analysis of Fig. 5 (bottom) to account for intermediate levels of innate immune activation in the context of combined therapy. We vary the levels of innate immune response activation from 20% to 100% (a, b, c, d, and e). Bacterial density is calculated after 96 h of treatment. Colored regions represent bacterial presence while white regions indicate infection clearance. Phage and antibiotic are administered 2 h after infection. Antibiotic levels vary from 0.1× to 10× MIC (MIC of ciprofloxacin = 0.014 μg/ml). Initial bacterial and phage density are *B*_0_ = 7.4 × 10^7^ CFU/g and *P*_0_ = 7.4 × 10^8^ PFU/g, respectively. Download FIG S4, PDF file, 0.02 MB. Copyright © 2020 Rodriguez-Gonzalez et al. 2020 Rodriguez-Gonzalez et al. This content is distributed under the terms of the Creative Commons Attribution 4.0 International license.

We performed a further exploratory analysis of the combined therapy. We studied the effects of delay times on the application of the combined strategy, showing that therapeutic action is robust to delay times and fails irrespective of delay time when the immune system is compromised (Text S1; Fig. S5). We also performed a parameter sensitivity analysis (Text S1), showing that the combined strategy, when supplemented with the host immune response, is effective for a wide range of parameters (Fig. S6).

10.1128/mSystems.00756-19.1 TEXT S1 Robustness and sensitivity analysis of the combination therapy model. Download Text S1, PDF file, 0.1 MB. Copyright © 2020 Rodriguez-Gonzalez et al. 2020 Rodriguez-Gonzalez et al. This content is distributed under the terms of the Creative Commons Attribution 4.0 International license.

10.1128/mSystems.00756-19.7 FIG S5 Time delays in the application of the combined treatment. We extend our robustness analysis of Fig. 5 (bottom) to account for time delays in the start of the combined treatment. Phage and antibiotic were administered simultaneously 2, 4, 6, 8, and 10 h after the beginning of the infection in the presence (a to e) or absence (f to j) of innate immunity. Colored regions on the heatmaps indicate bacterial presence while white regions indicate infection clearance. Antibiotic levels vary from 0.1× to 10× MIC (MIC of ciprofloxacin = 0.014 μg/ml). Initial bacterial and phage density are *B*_0_ = 7.4 × 10^7^ CFU/g and *P*_0_ = 7.4 × 10^8^ PFU/g, respectively. Download FIG S5, PDF file, 0.1 MB. Copyright © 2020 Rodriguez-Gonzalez et al. 2020 Rodriguez-Gonzalez et al. This content is distributed under the terms of the Creative Commons Attribution 4.0 International license.

10.1128/mSystems.00756-19.8 FIG S6 Parameter sensitivity analysis results. We show the distribution of the fraction of complete elimination for two therapeutic regimes, A + P + I (blue) and A + P (red). We performed 1,000 runs using perturbed parameter sets (θ_per_) and calculated the fraction of bacterial elimination for the two regimes. Moreover, we show the fraction of complete elimination for A + P + I (blue square) and A + P (red triangle) using the reference parameter set (θ_ref_). Download FIG S6, PDF file, 0.1 MB. Copyright © 2020 Rodriguez-Gonzalez et al. 2020 Rodriguez-Gonzalez et al. This content is distributed under the terms of the Creative Commons Attribution 4.0 International license.

Finally, we note that these results derived from analysis of dynamics arising among extreme phenotypes. In reality, phage-sensitive strains may retain some sensitivity to antibiotics and antibiotic-sensitive strains can be infected at reduced levels by phage (7, 23, 24). Hence, we repeated the robustness analysis, using an extended model that incorporates quantitatively different levels of phage infectivity and antibiotic sensitivity of both strains (see Text S2). Partial resistance model outcomes are qualitatively consistent with previous outcomes of the extreme resistance model (contrast Fig. S7 with Fig. 5). Moreover, the bacterial dynamics of the partial resistance model are qualitatively similar to the dynamics arising among extreme phenotypes (contrast Fig. 3 with Fig. S8, bottom). Overall, our model analysis suggests that robust, curative success of phage-antibiotic combination therapy could be driven, in part, by a largely unrealized synergy with the immune response.

10.1128/mSystems.00756-19.2 TEXT S2 Partial resistance model, an extension of the combination therapy model. Download Text S2, PDF file, 0.1 MB. Copyright © 2020 Rodriguez-Gonzalez et al. 2020 Rodriguez-Gonzalez et al. This content is distributed under the terms of the Creative Commons Attribution 4.0 International license.

10.1128/mSystems.00756-19.9 FIG S7 Robustness analysis of the partial resistance model for different antimicrobial strategies. Bacteria grew for 96 h exposed to different antimicrobial strategies: antibiotic-only (a), antibiotic plus innate immunity (b), phage and antibiotic (c), and phage-antibiotic combination plus active innate immunity (d). The heatmaps show the bacterial density at 96 h postinfection. Colored regions represent bacterial persistence while the clearance of the infection is represented by white regions. For the partial resistance model, phage can infect *B_A_* with an infection constant of ϕδ*_P_*, being δ*_P_* = 0.1. Moreover, the phage-sensitive strain, *B_P_*, is slightly sensitive to the antibiotic with a MIC of 0.172* *μg/ml. We simulate different concentrations of ciprofloxacin (CP), using the MIC of CP for the *B_A_* strain as a reference (MIC = 0.014* μ*g/ml). Fold changes in MIC go from 0.1× MIC (0.014* μ*g/ml) to 10× MIC (0.14* μ*g/ml). Furthermore, bacterial composition of the inoculum ranges from 100% phage-sensitive bacteria (0% *B_A_*) to 100% antibiotic-sensitive bacteria (100% *B_A_*). Initial bacterial density and phage density (c and d) are *B*_0_ = 7.4 × 10^7^ CFU/g and *P*_0_ = 7.4 × 10^8^ PFU/g, respectively. Phage and antibiotic are administered 2 h after the beginning of the infection. Download FIG S7, PDF file, 0.04 MB. Copyright © 2020 Rodriguez-Gonzalez et al. 2020 Rodriguez-Gonzalez et al. This content is distributed under the terms of the Creative Commons Attribution 4.0 International license.

10.1128/mSystems.00756-19.10 FIG S8 Time series of the partial resistance model in presence and absence of host immune response. We simulate the combination of phage and antibiotic against a phage-sensitive (a) and an antibiotic-sensitive (b) bacterial inoculum in the absence of the immune response. Moreover, we simulate a within-host scenario where the combined therapy interacts with the immune response (purple dashed line) and phage-sensitive bacteria (c) or antibiotic-sensitive bacteria (d). Here, phage (blue dashed line) and antibiotic are administered 2 h after the infection. Initial conditions are *B*_0_ = 7.4 × 10^7^ CFU/g and *P*_0_ = 7.4 × 10^8^ PFU/g. The concentration of antibiotic (0.0350 μg/ml = 2.5× MIC for *B_A_* strain) is maintained constant during the simulation (data not shown). The simulation run was 96 h (4 days). Download FIG S8, PDF file, 0.04 MB. Copyright © 2020 Rodriguez-Gonzalez et al. 2020 Rodriguez-Gonzalez et al. This content is distributed under the terms of the Creative Commons Attribution 4.0 International license.

## DISCUSSION

We have developed a combination therapy model that combines phage and antibiotics against a mixed-strain infection of Pseudomonas aeruginosa. The model suggests that infection clearance arises from nonlinear synergistic interactions between phage, antibiotic, and innate immunity. Moreover, the infection clearance shows robustness to variations in the concentration of antibiotic, delays in the administration of the combined therapy, the bacterial composition of the inoculum, and model assumptions. In contrast, when innate immunity responses are removed (or severely reduced), then phage-antibiotic combination therapy is predicted to fail to eliminate the infection. This suggests that combined therapy may depend critically on immune response for resolving bacterial infections.

The *in silico* findings are consistent with qualitative, experimental outcomes *in vitro* and *in vivo*. For example, one of our main results states that phage-antibiotic combined therapy has a greater antimicrobial effect than single-phage or antibiotic therapies; this is consistent with several *in vitro* settings that show a greater bacterial density reduction for combined rather than single therapies (25–28). Moreover, additional studies explore the use of sublethal concentrations of antibiotics otherwise insufficient for controlling bacterial growth but efficient when combined with phage against diverse bacterial populations (25, 26, 28, 29). These findings are consistent with our *in silico* outcomes where pathogen clearance is observed at sub-MIC antibiotic levels in the combined therapy framework. Further work to compare model-based predictions to experiments will require moving beyond outcomes to high-resolution temporal data.

In connecting models to experiment, it is important to consider extending the model framework to a spatially explicit context. Spatial structure can be relevant therapeutically. For example, during chronic infections spatially organized bacterial aggregates of P. aeruginosa protect themselves against phage killing by producing exopolysaccharides (30). Furthermore, modeling efforts have shown that spatial structure affects the therapeutic success of phage therapy (31) and phage-antibiotic combination therapy (32). For example, structured environments limit phage dispersion and amplification, promoting bacterial survival and resistance acquisition (31, 32). Moreover, the heterogeneous distribution of antibiotic creates spatial refuges (of low or null antimicrobial presence) where bacteria survive and resistant mutants arise (32). The current model also neglects the complex features of immune response termination (33) and interactions with commensal microbes (34), both priority areas for future work.

In conclusion, the phage-antibiotic combination therapy model developed here describes efforts to explore how host immunity modulates infection outcomes. As we have shown, immune clearance of pathogens may lie at the core of the curative success of combination treatments. If so, this additional synergy may help to resolve the resistance problem and also guide use of sub-MICs of antibiotics. Besides reducing toxic side effects associated with high concentrations of antibiotics, sub-MICs can improve phage infectivity through morphological changes of the bacterial cell (9, 35, 36) or by not interfering with the phage replication cycle (25, 26). When combined in an immunocompetent context, we find that phage-antibiotic combination therapy is robust to quantitatively and qualitatively distinct resistance profiles. These findings reinforce findings that phage and antibiotics can be used to treat a certain class of MDR P. aeruginosa pathogens in patients (11, 37). Model results also highlight the role of the immune response in realizing curative success—which will be relevant to expanding combination theory for a range of clinical applications.

## MATERIALS AND METHODS

### Model simulation.

The numerical integration of the combination therapy model is carried out using ODE45 in Matlab. We obtain the temporal dynamics of two bacterial strains, phage, antibiotic, and innate immune response. Moreover, we set an extinction threshold of 1 g^−1^; hence, when *B_P_* or *B_A_* densities are ≤1 CFU/g at any time during the simulation, we set their densities to 0 CFU/g. We run all the simulations for 96 h (4 days).

### Robustness analysis.

We perform a robustness analysis of the phage-antibiotic combination therapy model by varying its initial conditions. We vary the concentration of antibiotic from sub-MICs (0.1× MIC) to above MICs (10× MIC), using the MIC of ciprofloxacin (0.014* *μg/ml) for the PAPS phage-resistant strain as a reference (11). Moreover, we vary the bacterial composition of the inoculum by increasing the bacterial density of one strain (e.g., *B_A_*) by 5% and decreasing the density of the other by 5%. Then, we select a pair of initial conditions and run the model 96 h. Finally, we calculate total bacterial density, *B*_total_ = *B_A_* + *B_P_*.

### Parameter estimation.

The parameter values used in the simulations of the combination therapy model are shown in Tables 2 and 3. Most of the parameter estimation was carried out in previous work (see “Parameter Estimation” section in reference 5), supplemented by parameters associated with functions describing the pharmacodynamics and pharmacokinetics of ciprofloxacin (18, 38).

**TABLE 2:** Microbiology and phage-associated parameter values

Parameter of model	Value	Source from which estimated
Combination therapy model		
*r_P_*, maximum growth rate of phage-sensitive (antibiotic- resistant) bacteria	0.75 h^−1^	P. aeruginosa murine pneumonia model (40)
*K_C_*, carrying capacity of bacteria	1 × 10^10^ CFU/g	Assuming ∼4 times above the typical bacterial density (2.4 × 10^9^ CFU/g) in wild-type mice 24 h postinfection
β, burst size of phage	100	Estimated from reference 5
ω, decay rate of phage	0.07 h^−1^	Estimated from reference 5
ε, killing rate parameter of immune response	8.2 × 10^−8^ g/(h cell)	Set such that ε*K_I_* gives the maximum granulocyte killing rate (40)
*α*, maximum growth rate of immune response	0.97 h^−1^	Fitting of neutrophil recruitment data (41)
*K_I_*, maximum capacity of immune response	2.4 × 10^7^ cell/g	Fitting of neutrophil recruitment data (41)
*K_I_*, maximum capacity of immune response (immunodeficient mice)	Same as *I*_0_	No innate immune activation
*K_D_*, bacterial concentration at which immune response is half as effective	4.1 × 10^7^ CFU/g	Corresponds to lethal dose of about 5.5 × 10^6^ CFU/lungs
*K_N_*, bacterial concentration when immune response growth rate is half its maximum	10^7^ CFU/g	*In vitro* data of TLR5 response to PAK strain (42)
*B*_0_, initial bacterial density (in presence or absence of the innate immune response)	7.4 × 10^7^ CFU/g	Total inoculum of 10^7^ CFU
*P*_0_, initial phage dose (in presence or absence of the innate immune response)	7.4 × 10^8^ PFU/g	Total phage dose of 10^8^ PFU
*I*_0_, initial immune response	2.7 × 10^6^ cell/g	Fitting of neutrophil recruitment data (41)
*I*_0_, initial immune response (immunodeficient mice)	0 cell/g	Assuming no primary innate immunity
HM model		
$\tilde{\phi }$, nonlinear phage adsorption rate	5.4 × 10^−8^ (g/PFU)^γ^ h^−1^	Estimated from reference 5
γ, power law exponent in phage infection rate	0.6	Estimated from reference 5
PS model		
ϕ, linear phage adsorption rate	5.4 × 10^−8^ (g/PFU) h^−1^	Estimated from reference 5
*P_C_*, phage concentration at which phage infection rate is half saturated	1.5 × 10^7^ PFU/g	Estimated from reference 5
LI model		
ϕ, linear phage adsorption rate	5.4 × 10^−8^ (g/PFU) h^−1^	Estimated from reference 5

**TABLE 3:** Additional parameter values associated with the effects of antibiotics

Antibiotic (ciprofloxacin) parameter	Value	How calculated
κ_kill_, maximum antibiotic killing rate	18.5 h^−1^	Fitting an *E*_max_ model to antibiotic kill curves (18)
EC_50_, concentration of antibiotic at which the killing rate is half its maximum	0.3697 μg/ml	Calculated using the MIC of ciprofloxacin for the phage-resistant PAPS strain (7)
${\text{EC}}_{B_{P}}$, concentration of antibiotic at which the killing rate is half its maximum	4.070 μg/ml	Calculated using the MIC of ciprofloxacin for the phage-sensitive PAPS strain (7)
*H*, Hill coefficient	1	From reference 18
MIC of ciprofloxacin for P. aeruginosa PAPS phage-resistant strain	0.014 μg/ml	From reference 7
MIC of ciprofloxacin for P. aeruginosa PAPS phage-sensitive strain	0.172 μg/ml	From reference 7
θ, antibiotic elimination rate from serum samples	0.53 h^−1^	Estimated from antibiotic concentration-vs-time curves; concentration of ciprofloxacin was measured in serum samples of P. aeruginosa-infected mice (38)
Antibiotic-sensitive bacterial parameters		
*μ_1_, probability of emergence of antibiotic-sensitive (phage-resistant) mutants per cellular division	2.85 × 10^−8^	Estimated from experimental measurements (39)
μ_2_, probability of emergence of phage-sensitive (antibiotic-resistant) mutants per cellular division	2.85 × 10^−8^	Approximated to the estimates from reference 39
*r_A_*, maximum growth rate of antibiotic-sensitive (phage-resistant) bacteria	0.675 h^−1^	10% tradeoff between resistance against phage and growth rate (43)

The pharmacodynamics of ciprofloxacin (CP) is described by the following maximum effect (*E*_max_) model (18):

$$\kappa_{\text{kill}}\frac{A^{H}}{{\text{EC}}_{50}^{H}+A^{H}}.$$

where κ_kill_ represents the maximum killing rate of the antibiotic, EC_50_ is the antibiotic concentration at which the antibiotic killing rate is half its maximum, and *H* is a Hill coefficient. The values of the parameters are obtained using *in vitro* growth curves of P. aeruginosa at different concentrations of CP (18). The elimination rate of the antibiotic, *θ*, is estimated from levels of clearance of CP from serum samples of mice infected with P. aeruginosa (38). The EC_50_ parameter value is adjusted in our model to consider the MIC of CP for the PAPS reference strain (7).

The probabilities of producing a mutant strain per cell division, μ_1_ and μ_2_, are obtained from reference 39, where *μ*_1_ is the probability of producing a phage-resistant (antibiotic sensitive) mutant per cell division and *μ*_2_ is the probability of generating a phage-sensitive (antibiotic-resistant) mutant per cell division.

To account for partially resistant strains, we extend our combination therapy model (Text S2, equations S21 to S25) and include the parameters ${\text{EC}}_{B_{P}}$ and δ*_P_*. ${\text{EC}}_{B_{P}}$ is the half-saturation constant of the antibiotic killing function and modulates the level of antibiotic resistance for *B_P_*. The parameter was calculated based on the MIC of CP for the PAPS phage-sensitive strain (7). Moreover, for modulating the level of resistance to phage infection [*F*(*P*)] of *B_A_*, we use the parameter δ*_P_* < 1 (Table 4).

**TABLE 4:** Phage adsorption rate of phage-sensitive and phage-resistant Pseudomonas aeruginosa strains

Phage adsorption rate (%) for strain type:		Fold decrease	Reference
Phage sensitive	Phage resistant		
31.3	9.6	3.2	23
73.7	66.5–2.4	∼1.1 to ∼30	44
87.2	42.8	2	44
92.7	55.3 and 42.8	1.6 and 2	24

### Data availability.

The code used to simulate the phage-antibiotic combination therapy model and generate the main figures as well as the supplemental material figures can be found in the GitHub repository at https://github.com/WeitzGroup/phage_antibiotic.
