# Supplementary material for: Quantitative Models of Phage-Antibiotic Combination Therapy
Source: mSystems. 2020 Feb 4;5(1):e00756-19. doi: 10.1128/mSystems.00756-19 (PMC7002117; doi:10.1128/mSystems.00756-19)
Supplement: FIG S1 [file mSystems.00756-19-sf001.pdf]

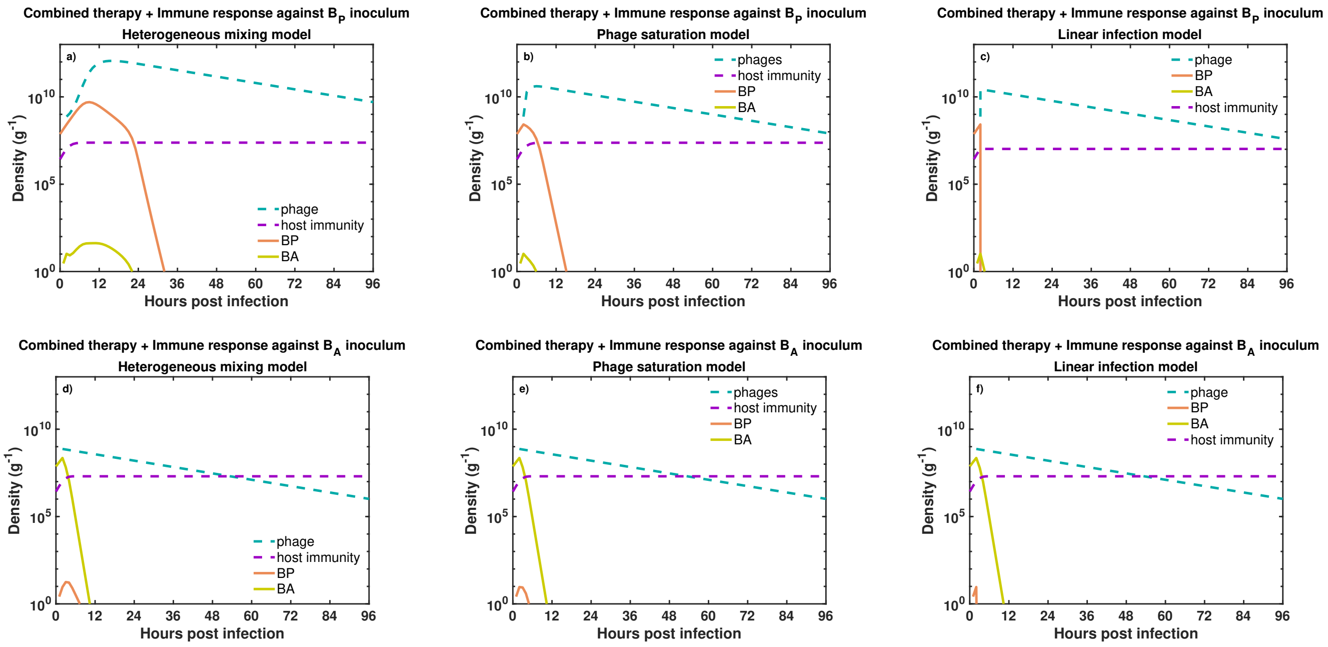
Combined therapy + Immune response against B P inoculum
Heterogeneous mixing model
a)
phage
host immunity
BP
BA
Combined therapy + Immune response against B P inoculum
Phage saturation model
b)
phages
host immunity
BP
BA
Combined therapy + Immune response against B P inoculum
Linear infection model
c)
phage
BP
BA
host immunity
Combined therapy + Immune response against B A inoculum
Heterogeneous mixing model
d)
phage
host immunity
BP
BA
Combined therapy + Immune response against B A inoculum
Phage saturation model
e)
phages
host immunity
BP
BA
Combined therapy + Immune response against B A inoculum
Linear infection model
f)
phage
BP
BA
host immunity
Density (g-1)
10^10
10^5
10^0
0 12 24 36 48 60 72 84 96
Hours post infection
