# Supplementary figures and images for: Quantitative Models of Phage-Antibiotic Combination Therapy
Source: mSystems. 2020 Feb 4;5(1):e00756-19. doi: 10.1128/mSystems.00756-19 (PMC7002117; doi:10.1128/mSystems.00756-19)

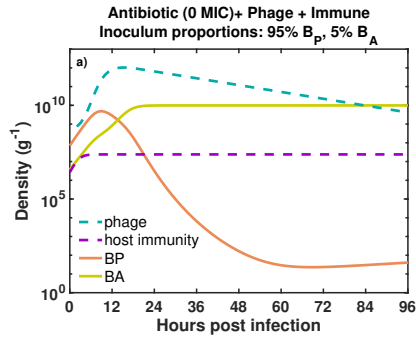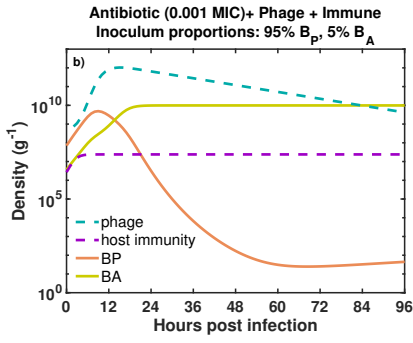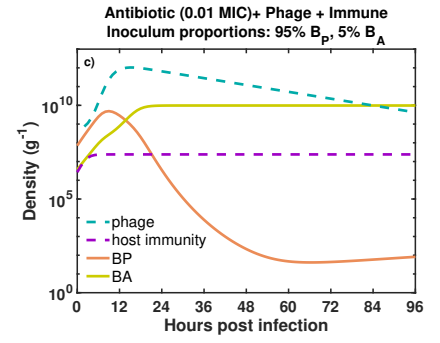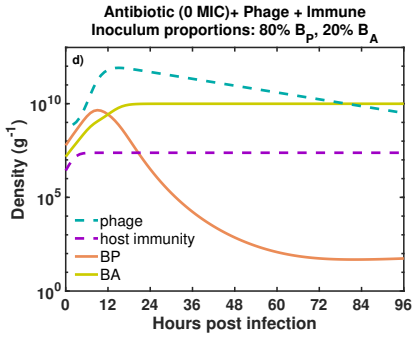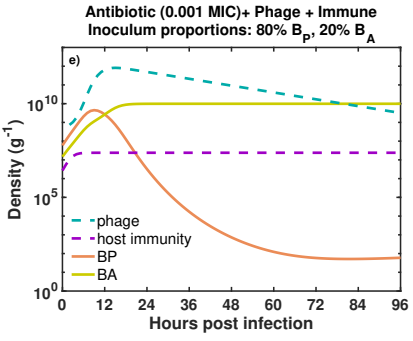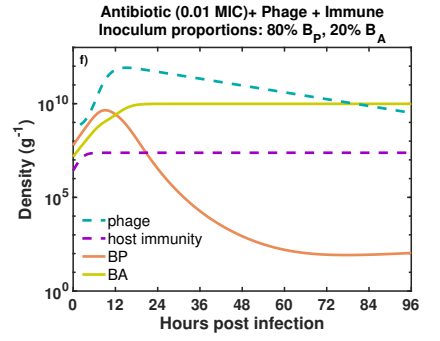

Supplement: FIG S2 [file mSystems.00756-19-sf002.pdf]

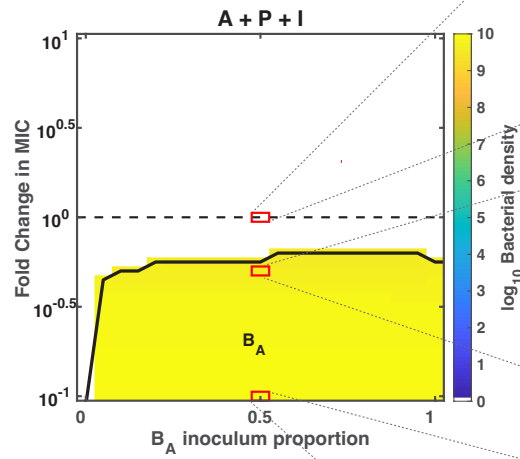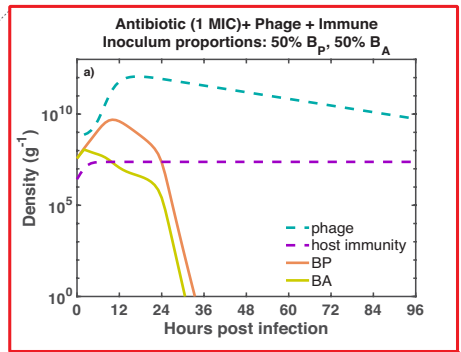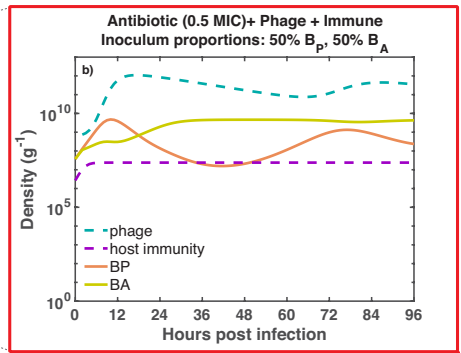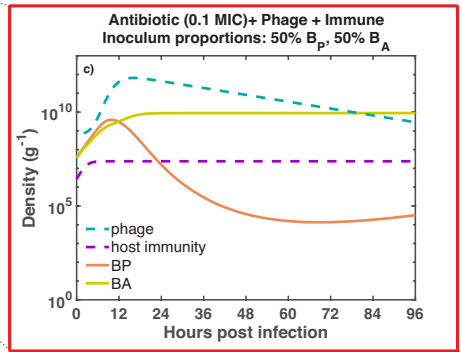

Supplement: FIG S3 [file mSystems.00756-19-sf003.pdf]

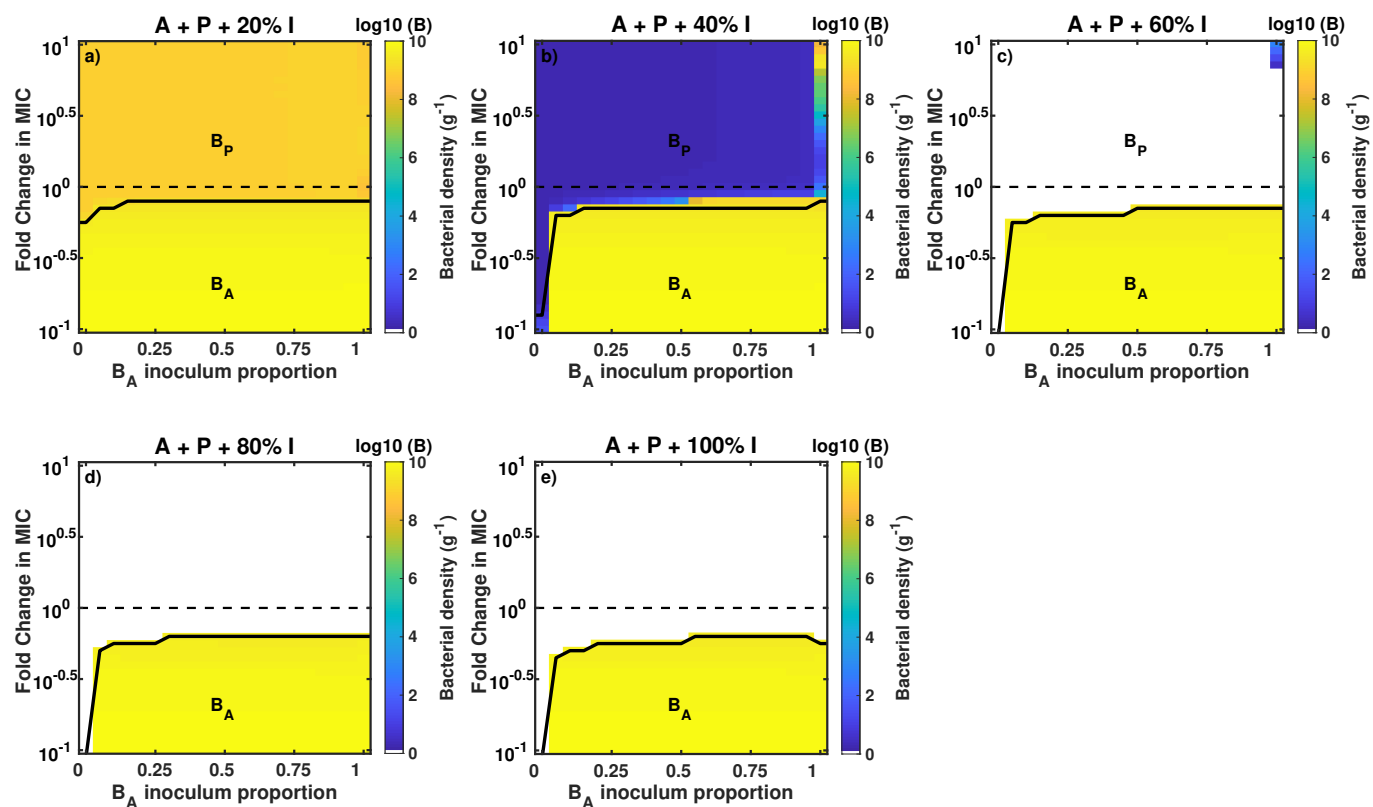

Supplement: FIG S4 [file mSystems.00756-19-sf004.pdf]

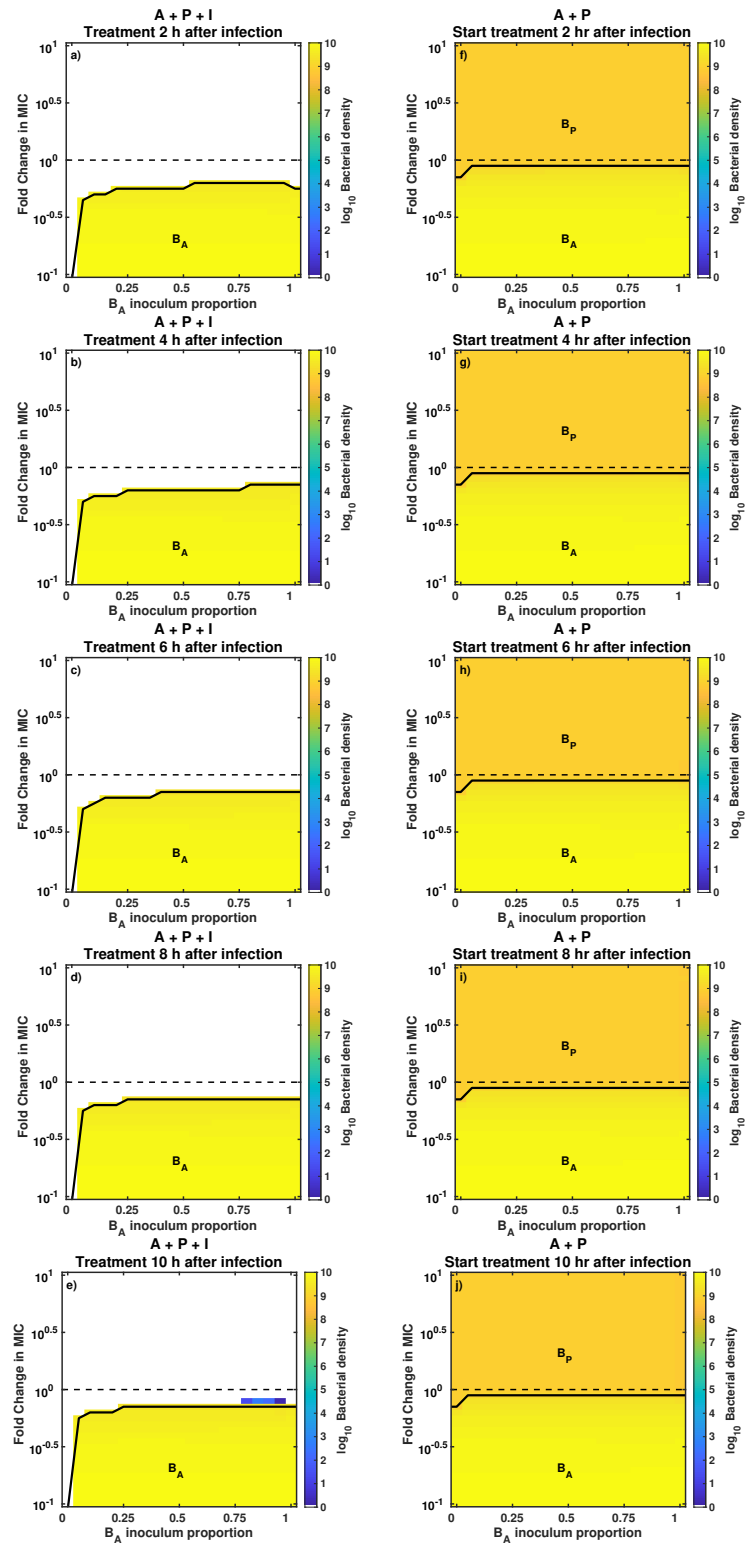

Supplement: FIG S5 [file mSystems.00756-19-sf005.pdf]

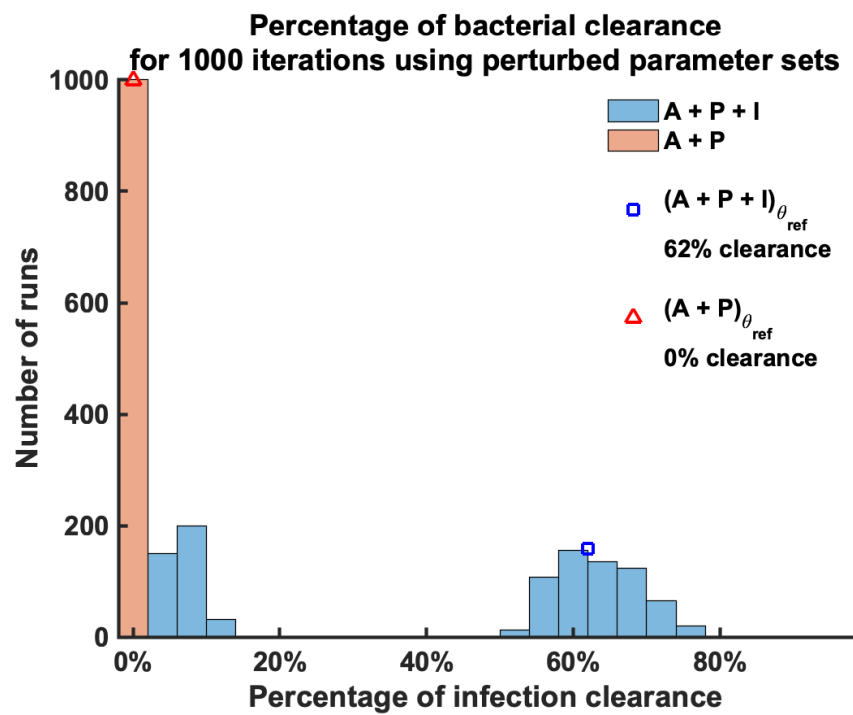

Supplement: FIG S6 [file mSystems.00756-19-sf006.pdf]

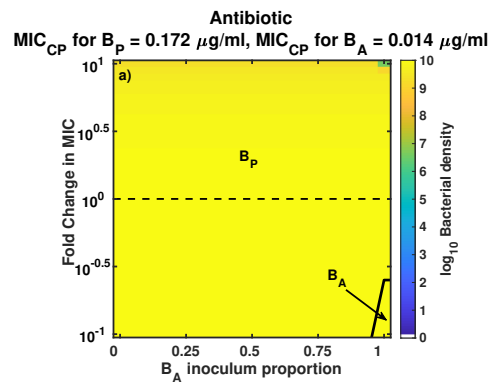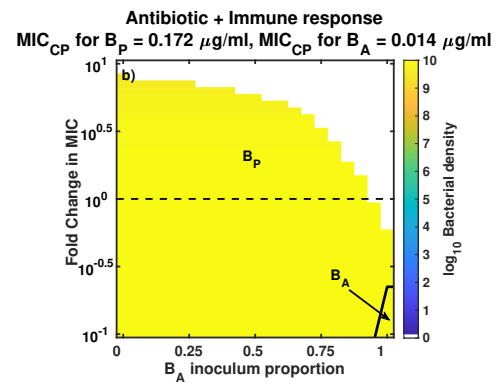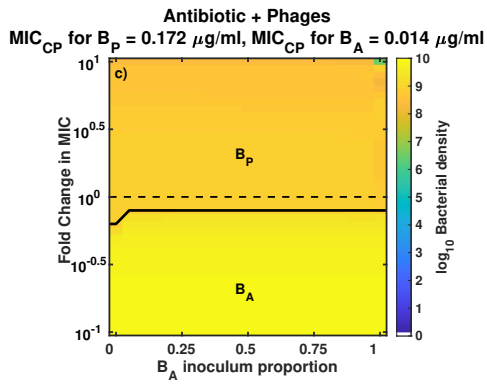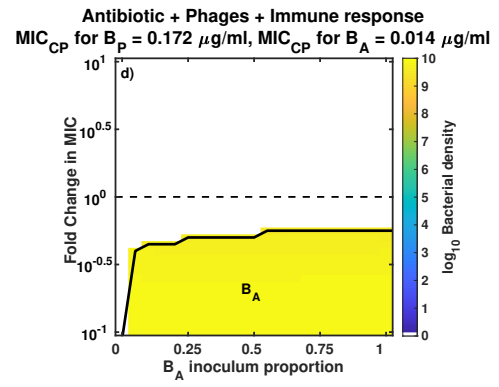

Supplement: FIG S7 [file mSystems.00756-19-sf007.pdf]

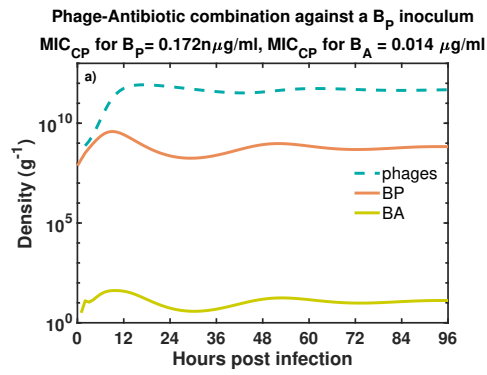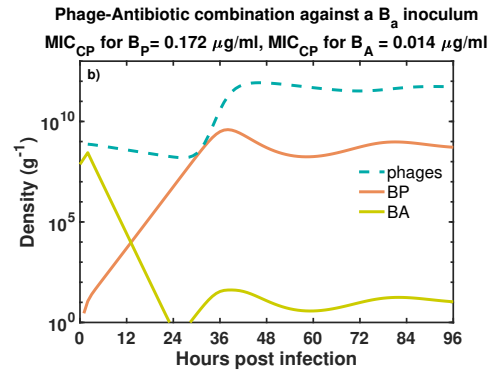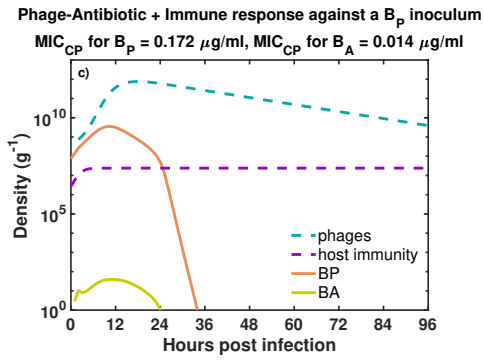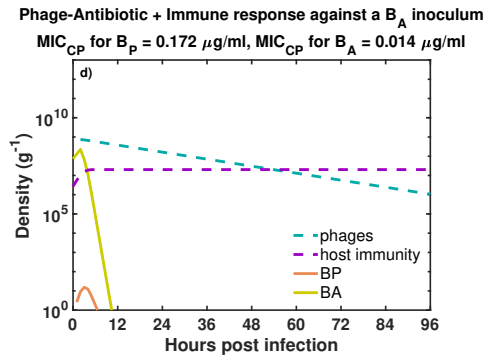

Supplement: FIG S8 [file mSystems.00756-19-sf008.pdf]
