# Supplementary material for: Quantitative Models of Phage-Antibiotic Combination Therapy
Source: mSystems. 2020 Feb 4;5(1):e00756-19. doi: 10.1128/mSystems.00756-19 (PMC7002117; doi:10.1128/mSystems.00756-19)
Supplement: TEXT S1 [file mSystems.00756-19-s0001.pdf]

Text S1: Robustness and sensitivity analysis of the combination therapy model

We performed a robustness analysis of the combined therapy model, where we varied the initial conditions such as concentration of antibiotics and composition of the bacterial inoculum (Fig. 5). Here, we analyze the effects of such variations on the bacterial dynamics. For example, the model predicts that when low or null concentrations of antibiotic are used against inoculum with non-trivial levels of antibiotic-sensitive bacteria B_A, the infection persists and B_A strain dominates after 96 hours (Fig. S2). On the other hand, infection clearance is obtained once we increase the concentration of the antibiotic, e.g., close to the MIC (Fig. S3-A), indicating that higher concentrations are needed to eliminate the infection for inoculum with non-trivial levels of B_A.

We further explore the claim that a sufficient level of immune response is needed to achieve a robust infection clearance. Hence, we varied the levels of innate immune activation from 20% to 100% for the A + P + I regime and simulated the combined therapy model for 96 hours. We predict that at least 60% of immune activation is needed to achieve a robust pathogen clearance (Fig. S4). We also explored the effects of time delays on the combination therapy. Phage and antibiotics were applied simultaneously 2 to 10 hours after the beginning of the infection, obtaining qualitatively consistent outcomes for this range of time-delays (Fig. S5).

Finally, we performed a parameter sensitivity analysis to evaluate the robustness of our model outcomes to parameter variations. Because parameters are adapted from experimental data whenever possible, we allow parameters to vary up to 10% of their original values. Then, we focused on two therapeutic regimes, A + P + I and A + P, and asked what fraction of the domain range in MIC and B_A proportion led to complete elimination (white regions on A + P + I heatmaps); this value is 62% for A + P + I and 0% for A + P when the reference parameter set, θ_{ref}, is used. We iterate this process 1000 times using different perturbed parameter sets, θ_{per}, and obtain a distribution of the percentage of complete elimination for the two therapeutic regimes. We found that $\sim 2/3$ of the total runs led to a fraction of 50% or more complete elimination (Fig. S6) for the A + P + I regime while no elimination was observed for the A + P regime in 1000 iterations. Overall, the sensitivity analysis supports the claim that joint synergistic effects of phage, antibiotics, and the immune response occur for many (rather than a particular choice of) parameter sets.
