# Supplementary material for: Quantitative Models of Phage-Antibiotic Combination Therapy
Source: mSystems. 2020 Feb 4;5(1):e00756-19. doi: 10.1128/mSystems.00756-19 (PMC7002117; doi:10.1128/mSystems.00756-19)
Supplement: TEXT S2 [file mSystems.00756-19-s0002.pdf]

Text S2: Partial resistance model, an extension of the combination therapy model

The extended *in vivo* combination therapy model describes a system where two bacterial strains partially sensitive to both, phage and antibiotic, interact with an active host immune response:

$$\dot{B}_P = r_P B_P \left(1 - \frac{B_P + B_A}{K_C}\right)(1-\mu_1) - B_P F(P) + \mu_2 r_A B_A \left(1 - \frac{B_P + B_A}{K_C}\right)$$

$$- \kappa_{kill} \overbrace{\frac{A^H}{EC_{B_P}^H + A^H}}^{\text{Antibiotic killing}} B_P - \overbrace{\frac{\varepsilon I B_P}{1 + \frac{(B_P + B_A)}{K_D}}}^{\text{Immune Killing}}, \tag{S21}$$

$$\dot{B}_A = r_A B_A \left(1 - \frac{B_P + B_A}{K_C}\right)(1-\mu_2) + \mu_1 r_P B_P \left(1 - \frac{B_P + B_A}{K_C}\right) - \overbrace{\delta_P B_A F(P)}^{\text{Lysis}}$$

$$-\kappa_{kill} \frac{A^H}{EC_{50}^H + A^H} B_A - \overbrace{\frac{\varepsilon I B_A}{1 + \frac{(B_P + B_A)}{K_D}}}^{\text{Immune Killing}}, \tag{S22}$$

$$\dot{P} = \tilde{\beta} B_P F(P) + \overbrace{\delta_P \tilde{\beta} B_A F(P)}^{\text{Viral release from } B_A} - \omega P, \tag{S23}$$

$$\dot{I} = \overbrace{\alpha I \left(1 - \frac{I}{K_I}\right)\left(\frac{B_P + B_A}{B_P + B_A + K_N}\right)}^{\text{Immune stimulation}}, \tag{S24}$$

$$\dot{A} = A_I - \theta A \tag{S25}$$

In this model, the parameter EC_{B_P} modulates the level of antibiotic resistance of the phage-sensitive bacteria, B_P (Equation S21) and $0 \leq \delta_P \leq 1$ modulates the level of phage-resistance of B_A (Equation S22). Finally, we add a new viral release term in the phage dynamics that accounts for phage infection of antibiotic-sensitive bacteria (Equation S23).

We simulate the partial resistance model covering immunodeficient and immunocompetent states (Fig. S8 top and bottom, respectively). We applied the combined treatment against two different infection settings, in the first setting the inoculum consisted of exclusively phage-sensitive bacteria and the second inoculum was composed of exclusively antibiotic-sensitive bacteria. Our simulations predict that the combined treatment may fail to clear the infection under an immunodeficient state regardless of the bacterial composition of the inoculum. This result is consonant with our previous finding (compare Fig. S8-top with Fig. 4). On the other hand, the incorporation of an active innate immunity may facilitate the elimination of the infection regardless of the bacterial genotype, i.e., phage-sensitive (Fig. S8, bottom-left) or antibiotic-sensitive (Fig. S8, bottom-right).

The combined effect between phage, antibiotic, and innate immune response leads to a synergistic infection clearance (Fig. S7, bottom-right). Our results suggest that even for bacterial strains that remain partially sensitive to both phage and antibiotics, the presence of the host innate immunity is still necessary to clear the infection. Hence, the outcomes of the combination therapy model are robust to model extensions that account for partially resistant strains.
